# The Interpretation of Standard Cardiopulmonary Exercise Test Indices of Cardiac Function in Chronic Kidney Disease

**DOI:** 10.3390/jcm12237456

**Published:** 2023-12-01

**Authors:** Shanmugakumar Chinnappa, Ming-Chieh Shih, Yu-Kang Tu, Andrew Mooney

**Affiliations:** 1 Department of Nephrology, Doncaster and Bassetlaw Teaching Hospitals NHS Trust, Doncaster DN2 5LT, UK; 2 Leeds Institute of Cardiovascular and Metabolic Medicine (LICAMM), University of Leeds, Leeds LS2 9JT, UK; andrew.mooney2@nhs.net; 3 School of Medicine, National Tsing Hua University, Hsinchu 300044, Taiwan; 4 Institute of Epidemiology and Preventive Medicine, National Taiwan University, Taipei 10617, Taiwan; yukangtu@ntu.edu.tw; 5 Department of Nephrology, Leeds Teaching Hospitals NHS Trust, Leeds LS9 7TF, UK

**Keywords:** chronic kidney disease, cardiopulmonary exercise test, heart failure, oxygen pulse, anaerobic threshold

## Abstract

Background and Aims: As there is growing interest in the application of cardiopulmonary exercise test (CPX) in chronic kidney disease (CKD), it is important to understand the utility of conventional exercise test parameters in quantifying the cardiopulmonary fitness of patients with CKD. Merely extrapolating information from heart failure (HF) patients would not suffice. In the present study, we evaluated the utility of CPX parameters such as the peak O_2_-pulse and the estimated stroke volume (SV) in assessing the peak SV by comparing with the actual measured values. Furthermore, we compared the anaerobic threshold (AT), peak circulatory power, and ventilatory power with that of the measured values of the peak cardiac power (CPO_peak_) in representing the cardiac functional reserve in CKD. We also performed such analyses in patients with HF for comparison. Method: A cross sectional study of 70 asymptomatic male CKD patients [CKD stages 2–5 (pre-dialysis)] without primary cardiac disease or diabetes mellitus and 25 HF patients. A specialized CPX with a CO_2_ rebreathing technique was utilized to measure the peak cardiac output and peak cardiac power output. The peak O_2_ consumption (VO_2peak_) and AT were also measured during the test. Parameters such as the O_2_-pulse, stroke volume, arteriovenous difference in O_2_ concentration [C(a-v)O_2_], peak circulatory power, and peak ventilatory power were all calculated. Pearson’s correlation, univariate, and multivariate analyses were applied. Results: Whereas there was a strong correlation between the peak O_2_-pulse and measured peak SV in HF, the correlation was less robust in CKD. Similarly, the correlation between the estimated SV and the measured SV was less robust in CKD compared to HF. The AT only showed a modest correlation with the CPO_peak_ in HF and only a weak correlation in CKD. A stronger correlation was demonstrated between the peak circulatory power and CPO_peak_, and the ventilatory power and CPO_peak_. In HF, the central cardiac factor was the predominant determinant of the standard CPX-derived surrogate indices of cardiac performance. By contrast, in CKD both central and peripheral factors played an equally important role, making such indices less reliable markers of cardiac performance per se in CKD. Conclusion: The results highlight that the standard CPX-derived surrogate markers of cardiac performance may be less reliable in CKD, and that further prospective studies comparing such surrogate markers with directly measured cardiac hemodynamics are required before adopting such markers into clinical practice or research in CKD.

## 1. Introduction

There has been a significant revival in the application of physical exercise as a diagnostic tool and as therapy per se in chronic kidney disease (CKD) and end stage renal disease. The diagnostic application includes assessment of cardiovascular fitness and risk stratification before renal transplant surgery, and the therapeutic application takes the form of exercise training [1,2]. All these applications require objective measures of exercise capacity and a clear understanding of the scope and limitations of these measures. The gold standard technique to measure exercise capacity is a cardiopulmonary exercise test (CPX). However, there are very few studies evaluating the utility of CPX-derived indices of cardiovascular fitness in CKD.

The crux of CPX testing is the Fick’s equation [VO_2_ = SV × HR × C(a-v)O_2_], where VO_2_ is the O_2_ consumption, SV is stroke volume, HR is heart rate, and C(a-v)O_2_ is arteriovenous difference in the O_2_ concentration. Of the variables in the Fick’s equation, in a standard CPX only VO_2_ and HR are measured at peak exercise. Stroke volume and C(a-v)O_2_ are not measured. Peak O_2_ consumption (VO_2peak_) or maximal aerobic capacity is widely used as a marker of cardiovascular fitness in the general population and in patients with heart failure (HF). Along with VO_2peak_, the indices O_2_-pulse (VO_2_/HR), a surrogate of SV, and VO_2_ at the anaerobic threshold (AT), are also widely used as surrogate markers of cardiovascular fitness. An important assumption in these applications is that impaired cardiac function is the predominant determinant of impaired exercise capacity. In the above applications, C(a-v)O_2_ is assumed to be a constant.

However, our recently published work demonstrated that peripheral non-cardiac factors are the major determinants of exercise capacity in CKD [3]. This contrasts with heart failure where, not surprisingly, central cardiac factors are the predominant determinants of exercise capacity. Exploration of the determinants of exercise capacity was made possible by the novel techniques of measuring non-invasive cardiac output (NICO) during CPX in addition to measuring VO_2_ [4,5,6]. This simultaneous measurement of cardiac output and VO_2_ enables computation of C(a-v)O_2_ using the Fick’s equation. This in turn enables a thorough evaluation of the various determinants of exercise capacity.

In the present study, we evaluated the utility of standard CPX-derived surrogate indices of cardiac performance by comparing with the actual measured values. In addition to testing patients with CKD, we also performed such analyses in patients with heart failure to bear out the distinction between the performance of these surrogate indices in two different settings: one where the cardiac factors are the predominant determinants of exercise capacity (i.e., HF), and the other where the peripheral factors play a major role (i.e., CKD). The surrogate indices evaluated in the study includes the peak O_2_-pulse, estimated peak SV and AT. In addition, we also evaluated surrogate indices of the peak cardiac performance and cardiac functional reserve such as the peak circulatory power and the peak ventilatory power with that of the measured parameter, the peak cardiac power (CPO_peak_) [6].

## 2. Methods

### 2.1. Study Subjects

In this cross-sectional study of adult patients, 70 asymptomatic male CKD patients [stages 2–5 (pre-dialysis)] and 25 age-matched male HF patients (NYHA Class II and III) were recruited from a tertiary UK center. Exclusion criteria for the CKD patients comprised an inability or contraindication to exercise on a treadmill; diabetes mellitus; any known cardiac disease (ischemic, arrhythmic or valvular); limitation of exercise ability due to overt musculoskeletal, cardiovascular, pulmonary, hepatic, neurological, or other non-renal medical disorders. 

### 2.2. Study Investigations

Blood test: Venous blood samples were taken at the time of recruitment to assay serum creatinine, urea, and hemoglobin. Estimated glomerular filtration rate (eGFR) was calculated using the 4-variable modification of diet in renal disease MDRD formula [7].

Cardiopulmonary exercise test (CPX): The patients underwent a specialized CPX on a treadmill to measure the VO_2peak_ and peak cardiac output (CO) simultaneously, and compute C(a-v)O_2_ using the Fick’s equation. The peak cardiac output was measured non-invasively using CO_2_ rebreathing method (Medgraphics Corp., St. Paul, MN, USA) according to previously described methodology [3]. The peak O_2_ consumption and peak cardiac output were determined non-invasively during maximal cardiopulmonary exercise (CPX) testing.

- Resting measures: The O_2_ consumption, CO_2_ production, respiratory rate, and cardiac output at rest were measured using a Medgraphics CardiO_2_ Analytic System (Medgraphics Corp., St. Paul, MN, USA). Resting cardiac output was calculated using the Collier CO_2_ rebreathing method [8,9]. The Collier’s equilibration method has been shown to have good correlation with thermodilution techniques at rest [10] and is easy to use, and therefore it was utilized for resting measurements.
- Determination of exercise capacity (VO_2peak_): Subjects then underwent an incremental exercise test on a treadmill according to a standard Bruce protocol (or modified Bruce protocol for HF patients). The speed and incline of the treadmill were increased every three minutes according to the protocol until the subjects reached volitional exhaustion. Throughout the treadmill test, O_2_ consumption, CO_2_ production, end-tidal partial pressure of CO_2_, tidal ventilation, and respiratory rate were measured using breath-to-breath analysis. Ventilatory (“anaerobic”) threshold was measured by the V-slope method [11]. A 12-lead ECG was monitored throughout, and the subject’s heart rate (HR) was obtained from this. Blood pressure was measured at every stage of the CPX test.
- Determination of peak cardiac output: A second treadmill test was performed after a rest period of at least 40 minutes. The first treadmill test also served as a familiarization step. The speed and incline of the treadmill were adjusted manually. The subjects exercised on the treadmill to 95% of their VO_2peak_ as established in the incremental exercise test. Two or three cardiac output measurements were made using the Defare’s CO_2_ rebreathing method [12]. The Defare’s method was chosen because this method has been shown to correlate well with cardiac output obtained with thermodilution techniques during exercise [13]. The formulae used in the study are listed in Table 1. The blood pressure was measured using a sphygmomanometer after each determination of cardiac output.

### 2.3. Statistical Analysis

Independent sample *t*-test was utilized to compare anthropometrics, biochemistry, and CPX parameters between CKD and HF patients. Pearson’s correlation was used to evaluate the association between the surrogate indices and the measured values of peak cardiac performance. Univariate and multivariate regression was used to evaluate the determinants of surrogate indices of cardiac performance. Bland–Altman analysis was utilized to evaluate the agreement between the estimated and measured stroke volume. Normality of data was verified using normal Q-Q plots and numerical methods (Shapiro—Wilk test). All data were normally distributed. SPSS 25 (IBM, Armonk, NY, USA) statistics software was used in the analysis. A *p*-value of <0.05 was considered significant. Results are presented as mean ± SD.

## 3. Results

### 3.1. Patient Characteristics

The mean age of CKD and HF patients were 48.4 ± 12.6 and 49.4 ± 14.6 years respectively. The CKD patients included the spectrum of CKD from stages 2 to 5 (pre-dialysis). There were 21 patients with CKD stages 2–3a, 27 patients with CKD stages 3b–4, and 22 patients with CKD stage 5. The CKD cohort had a wide range of eGFR (6 mL/min to 88.5 mL/min) and hemoglobin (9.3 g/dL to 16.7 g/dL) ensuring that correlation and linear regression analyses were not limited by range restriction. All participants performed exercise to volitional exhaustion. The CKD and HF patients had a mean respiratory exchange ratio of 1.16 ± 0.09 and 1.10 ± 0.29, a peak VO_2_ of 2.66 ± 0.57 and 1.61 ± 0.37 L/min, and an AT of 1.81 ± 0.47 and 1.11 ± 0.35 L/min, respectively. Patient characteristics are presented in Table 2.

### 3.2. Association between the Peak SV and Peak O_2_-Pulse

**Figure 1:**
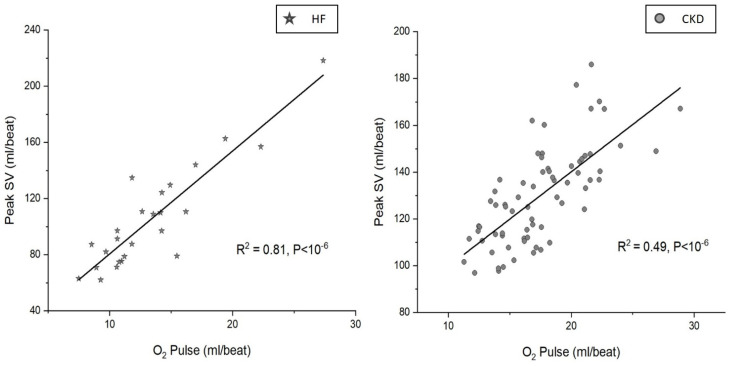
Association between peak O_2_ pulse and measured peak stroke volume in heart failure and chronic kidney disease. Peak O_2_ pulse shows a stronger association with peak SV in HF compared to CKD. SV: stroke volume, HF: heart failure, CKD: chronic kidney disease.

The association between the measured peak SV and the peak O_2_-pulse in CKD compared to HF is shown in Figure 1.

In HF, there was a stronger association between the measured peak SV and the peak O_2_-pulse (R^2^ = 0.81, *p* < 10^−6^) compared to CKD (R^2^ = 0.49, *p* < 10^−6^). Multiple regression analysis showed that in HF, SV (β = 1.03, *p* < 10^−3^) was the predominant determinant of O_2_-pulse, and C(a-v)O_2_ (β = 0.44, *p* < 10^−3^) played a less significant role. However, in CKD both SV (β = 0.78, *p* < 10^−3^) and C(a-v)O_2_ (β = 0.70, *p* < 10^−3^) were significant determinants of O_2_-pulse.

### 3.3. Association between the Estimated and Measured Peak SV

**Figure 2:**
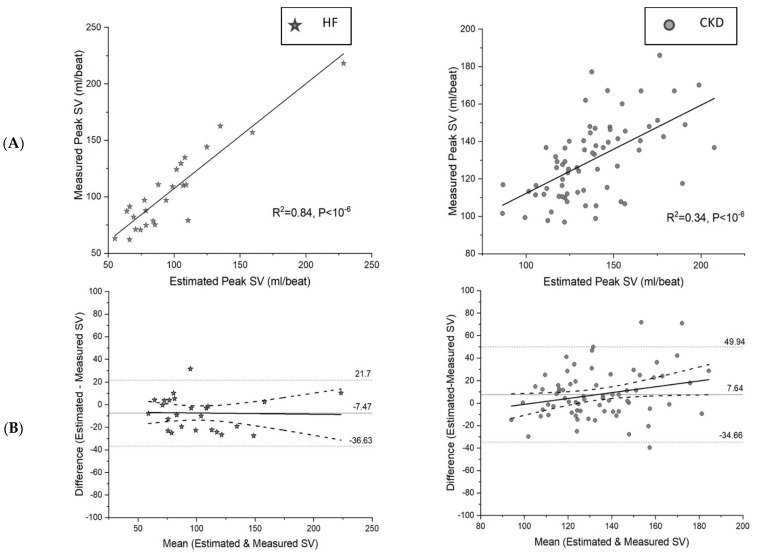
Association between estimated stroke volume and measured peak stroke volume in heart failure and chronic kidney disease. Estimated peak SV shows a stronger association with measured peak stroke volume in HF compared to CKD (**A**). The Bland–Altman plot shows wider limits of agreement in CKD with a statistically significant regression slope implying that estimated SV measurements can be imprecise in CKD (**B**). SV: stroke volume, HF: heart failure, CKD: chronic kidney disease.

The association between the estimated and measured peak SV in CKD was weaker (R^2^ = 0.34, *p* < 10^−6^) compared to that of HF (R^2^ = 0.84, *p* < 10^−6^) (Figure 2A). The Bland–Altman plot (Figure 2B) illustrates the agreement between the estimated and measured peak SV. The plot for the CKD group shows that, on average, the SV was overestimated [average bias = 7.64 mL (95% CI 2.50~12.79 units), *p* = 0.004].

The estimated boundaries for 95% of the differences between the estimated SV and the measured SV were 49.94 ml (95% CI 41.10–58.78) and −34.66 mL (95% CI −43.49–−25.82). The wide boundaries indicate that the estimated SV can be very imprecise and should be interpreted with caution. The regression slope was positive and statistically significant [slope = 0.26, (95% CI 0.01–0.51), *p* = 0.038], which implied that the positive bias of the estimated SV increased with the true SV. For the HF group, the SV was underestimated on average [average bias = −7.47 units (95% CI −13.61–−1.32 units), *p* = 0.017]. The estimated boundaries for 95% of the differences were 21.70 units (95% CI 11.06–32.34 units) and −36.63 units (95% CI −47.27–−25.99 units). While the boundaries were still wide, the standard deviation in the HF group was significantly smaller than in the CKD group (*p* = 0.044). The regression slope was not statistically significant [slope = 0.011 (95% CI −0.19–0.17), *p* = 0.90].

### 3.4. Association between the AT and Peak Cardiac Power (CPO_peak_)

**Figure 3:**
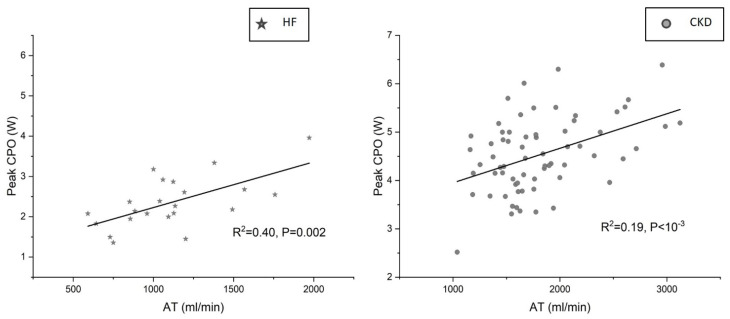
Association between anaerobic threshold and peak cardiac power in heart failure and chronic kidney disease. AT shows only a modest association with CPO_peak_ in CKD compared to HF. HF: heart failure, CKD: chronic kidney disease, AT: anaerobic threshold, CPO: cardiac power output.

The AT had only a modest association with the CPO_peak_ (R^2^ = 0.19, *p* < 0.05) in CKD compared to HF (R^2^ = 0.40, *p* < 0.05) (Figure 3).

The AT indexed to body weight had only a weak association with the CPO_peak_ (R^2^ = 0.098, *p* = 0.01) compared to HF (R^2^ = 0.31, *p* = 0.007). The AT expressed as a % of the VO_2peak_ did not have any correlation with the CPO_peak_ in CKD or HF. The independent predictors of the AT in CKD were hemoglobin concentration (β = 0.45, *p* < 10^−3^), estimated lean body mass (β = 0.33, *p* < 10^−3^), and age (β= −0.33, *p* < 10^−3^) together accounting for >50% variability in the AT (R^2^= 0.53, *p* < 10^−6^).

### 3.5. Association between the Peak Circulatory Power, Peak Ventilatory Power, and Peak Cardiac Power in CKD

**Figure 4:**
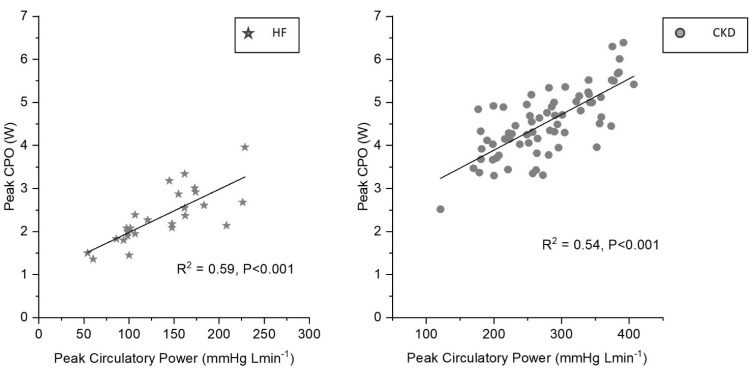
Association between peak circulatory power and peak cardiac power in heart failure and chronic kidney disease. Peak circulatory power shows a strong association with peak cardiac power in both HF and CKD. HF: heart failure, CKD: chronic kidney disease, CPO: cardiac power output.

The peak circulatory power showed a strong association with the CPO_peak_ in both CKD (R^2^ = 0.54, *p* < 0.01) and HF patients (R^2^ = 0.59, *p* < 0.01) (Figure 4).

The peak ventilatory power also showed good association with the CPO_peak_ in CKD (R^2^ = 0.41, *p* < 0.01) and HF (R^2^ = 0.30, *p* < 0.01).

## 4. Discussion

The study for the first time compared the utility of the CPX-derived surrogate markers of peak cardiac performance with the actual measured values in CKD and showed that such surrogate markers may be less reliable in CKD. There was a clear distinction between the performance of these surrogate markers in HF and in CKD.

The peak O_2_-pulse is a widely used surrogate marker of the peak SV in HF [15,16,17]. As the central cardiac factor is the predominant determinant of the VO_2peak_ in HF, the peak O_2_-pulse performs as a reliable indicator of the peak SV. This has in turn led to the formulation of the estimation equation of the peak SV using the peak O_2_-pulse in HF [18]. Our study results support such application in HF too. In our study, more than an 80% variation in the measured peak SV could be predicted by the peak O_2_-pulse and the estimated peak SV in HF. On the contrary, in CKD, as peripheral factors also play a significant role in determining exercise capacity, only 50% of the variation in the measured SV was predicted by the peak O_2_-pulse. Furthermore, only 34% of the variation in the measured SV was predicted by the estimated SV, making the parameters less reliable in CKD. The Bland–Altman plot reinforces this finding by demonstrating that the agreement between the estimated SV (derived from O_2_ pulse) and the measured SV is weaker in CKD compared to HF.

The AT marks the point in incremental exercise where O_2_ demand exceeds O_2_ supply and the skeletal muscle switches to anaerobic respiration to generate ATP. The O_2_ supply depends on the O_2_ carrying capacity of the blood and the cardiac output. Furthermore, the skeletal muscle properties such as the muscle mass, its vascularization, mitochondrial function, etc., would determine the O_2_ utilization and thereby the AT. In CKD, the O_2_ carrying capacity is impaired due to impaired cardiac function secondary to uremic cardiomyopathy [19,20,21] and uremic vasculopathy [22,23], and due to the impaired O_2_ carrying capacity of the blood secondary to anemia [24,25,26]. In addition, the O_2_ utilization is impaired due to the reduced capillary density of the skeletal muscles, sarcopenia, and mitochondrial dysfunction, collectively called as uremic skeletal myopathy [27,28,29]. It is therefore not surprising that, in CKD, the AT showed only a weak correlation with the peak cardiac power output, an objective measure of the peak cardiac performance and the cardiac functional reserve [30,31]. It is also pertinent to note that the hemoglobin level, estimated lean body mass, and age were the independent predictors of the AT in CKD accounting for >50% variability of the AT.

The AT is widely used as a marker of cardiovascular fitness for surgery in the general population. In CKD, there is growing desire to apply the AT in the assessment of cardiovascular fitness prior to renal transplant surgery [32]. Furthermore, the AT is also being considered as a marker of the peak cardiac performance and cardiac functional reserve in CKD. However, our study showed that the AT may not be a reliable indicator of the cardiac functional reserve in CKD. The results of the study highlight that one must be mindful of the fact several non-cardiac factors play a significant role as the determinants of such surrogate markers of cardiac performance in CKD. Therefore, CKD-specific studies may be required in the future to evaluate the application of the AT in the assessment of cardiovascular fitness for renal transplantation or other surgeries in patients with CKD.

The peak circulatory power and peak ventilatory power are relatively novel indices of the cardiac functional reserve calculated from standard CPX parameters such as the peak blood pressure, VO_2peak_ and ventilatory efficiency slope (V_E_/VCO_2_ slope). Such indices are shown to predict survival in patients with heart failure [33,34]. Our study showed that such surrogate indices demonstrated better correlation with CPO_peak_ than the AT. Further prospective studies are required to evaluate the application of such surrogate indices of the cardiac functional reserve in CKD.

The study has several strengths. A significant strength of the study is the use of HF patients as a positive control to highlight the distinction between the impaired exercise capacity due to predominantly cardiac factors and impairment due to multifactorial etiology as in CKD. Our unit has extensive experience of nearly three decades in the measurement of non-invasive cardiac output using the CO_2_ rebreathing technique [6,30], and all our study participants underwent CPX studies using the same standardized protocol. The study protocol with strict exclusion criteria is also a strength that helped minimize confounders that may affect exercise capacity other than CKD such as diabetes mellitus, cardiovascular diseases, respiratory disorders, etc. A limitation of the study is that we did not measure C(a-v)O_2_ directly. However, this is unlikely to have any impact on the study results as the calculated C(a-v)O_2_ from non-invasive cardiac output measurements and the directly measured C(a-v)O_2_ values using blood gas analysis were shown to have good agreement [4]. The assessments were limited to one gender to minimize confounders that arise because of gender and body composition on central hemodynamics and aerobic exercise capacity [5,24]. We employed treadmill exercise instead of bicycle ergometry because treadmill studies are shown to achieve higher VO_2peak_ [35] enhancing the probability of discrimination between health and disease states. Furthermore, treadmill exercise was a more familiar form of exercise in our cohort, minimizing the number of dropouts.

In conclusion, the standard CPX-derived surrogate markers of cardiac performance may be less reliable in CKD, and further prospective studies comparing such surrogate markers with the directly measured cardiac hemodynamics are required before adopting such markers into clinical practice or research in CKD.

## Figures and Tables

**Table 1:** List of formulae used in the study.

Parameter	Formula
VO_2_ (L/min)	CO × C(a-v)O_2_
C(a-v)O_2_ (mL/dL)	VO_2_/CO
O_2_ pulse (mL/beat)	VO_2_/HR
Estimated SV [14] (mL/beat)	$\frac{O_{2}Pulse}{Hb\;\times \;1.34\;\times \;O_{2}extraction(\%)}$
MAP (mmHg)	MAP = DBP + 0.412 (SBP − DBP)
CPO (W)	CO × MAP × 2.22 × 10^−3^
Peak Circulatory Power (mmHg L min^−1^)	MAP × VO_2peak_
Peak Ventilatory Power (mmHg)	$\frac{Peak\;SBP}{Ventilatory\;efficiency\;slope}$
Ventilatory efficiency slope	V_E_/VCO_2_
eLBM	(0.407 × Weight) + (0.267 × Height) − 19.2

VO_2_: O_2_ consumption, C(a-v)O_2_: arteriovenous O_2_ difference, HR: heart rate, Hb: hemoglobin, MAP: mean arterial pressure, DBP: diastolic blood pressure, SBP: systolic blood pressure, CO: cardiac output, CPO: cardiac power output, V_E_: minute ventilation, VCO_2_: CO_2_ production, LBM: estimated lean body mass (Boer’s formula).

**Table 2:** Patient Characteristics.

	CKD (n = 70)	HF (n = 25)	*p*-Value
Age (year)	48.4 ± 12.6	49.4 ± 14.6	NS
BMI (kg/m^2^)	27.8 ± 3.9	25.1 ± 3.2	<0.05
Hb (g/dL)	13.3 ± 1.8	14.4 ± 1.1	<0.05
eGFR (mL/min)	33.9 ± 23.5	69.3 ± 16.9	<0.05
Peak RER	1.16 ± 0.09	1.10 ± 0.29	NS
VO_2peak_ (L/min)	2.66 ± 0.57	1.61 ± 0.37	<0.05
AT (L/min)	1.81 ± 0.47	1.11 ± 0.35	<0.05
AT (mL/min/kg)	21.23 ± 5.18	14.61 ± 4.37	<0.05
Peak CO (L/min)	19.7 ± 2.6	12.5 ± 2.4	<0.05
Peak C(a-v)O_2_ (mL/dL)	13.4 ± 1.9	12.9 ± 2.2	NS
Peak HR (beats/min)	153.4 ± 19.9	126.9 ± 30.8	<0.05
Peak SV (mL/beat)	129.9 ± 20.7	105.1 ± 37.0	<0.05
Peak O_2_ pulse (mL/beat)	17.48 ± 3.62	13.35 ± 4.54	<0.05
Peak Circ pwr (mmHg L min^−1^)	277.9 ± 68.4	135.9 ± 47.9	<0.05
Ventilatory Power	5.36 ± 1.13	3.20 ± 1.21	<0.05
Peak CPO (W)	4.54 ± 0.77	2.34 ± 0.63	<0.05

BMI: body mass index, Hb: hemoglobin, eGFR: estimated glomerular filtration rate, RER: respiratory exchange ratio, VO_2_: oxygen consumption, AT: anaerobic threshold, CO: cardiac output, C(a-v)O_2_: peripheral O_2_ extraction, HR: heart rate, SV: stroke volume, Circ pwr: circulatory power, CPO: cardiac power output. *P*-value is for independent sample *t*-test.

## Data Availability

Data are contained within the article.
